# Association between comorbidities associated with diabetes and higher-level functional status in older patients with type 2 diabetes mellitus: a cross sectional study

**DOI:** 10.1007/s41999-024-00937-8

**Published:** 2024-02-10

**Authors:** Takuro Shoji, Kenta Kogure, Nagisa Toda, Mariko Hakoshima, Hisayuki Katsuyama, Hidekatsu Yanai, Satoshi Tokunaga, Korin Tateoka, Taishi Tsuji, Tomohiro Okura

**Affiliations:** 1 Department of Rehabilitation Medicine, National Center for Global Health and Medicine Kohnodai Hospital, Kohnodai 1-7-1, Ichikawa, Chiba 272-8516 Japan; 2 Graduate School of Comprehensive Human Sciences, Doctoral Program in Public Health, University of Tsukuba, Tsukuba, Ibaraki Japan; 3 Department of Diabetes, Endocrinology, and Metabolism, National Center for Global Health and Medicine, Kohnodai Hospital, Ichikawa, Japan; 4 Graduate School of Comprehensive Human Sciences, Doctoral Program in Physical Education, Health and Sport Sciences, University of Tsukuba, Tuskuba, Ibaraki Japan; 5 Institute of Health and Sport Sciences, University of Tsukuba, Tsukuba, Ibaraki Japan

**Keywords:** Depression, Frailty, Instrumental activities of daily living, Intellectual activity, Low muscle strength, Tokyo Metropolitan Institute of Gerontology Index of Competence

## Abstract

**Purpose:**

To investigate the association between comorbidities associated with diabetes and higher-level functional status as well as the relationship between comorbidities associated with diabetes and higher-level functional status in older patients with type 2 diabetes mellitus who have better social networks.

**Methods:**

Participants were outpatients with type 2 diabetes aged ≥ 65 years, excluding individuals with severe cardiovascular or respiratory illness, hyperglycaemic crisis, type 1 diabetes, or diabetic foot. The Tokyo Metropolitan Institute of Gerontology Index of Competence (TMIG-IC) was used to evaluate the higher-level functional status. A TMIG-IC score of ≤ 9, instrumental activities of daily living (IADL) ≤ 4, intellectual activity or social role ≤ 3 were defined as decline in higher-level functional status. The comorbidities investigated included peripheral neuropathy, retinopathy, nephropathy, cognitive impairment, depression, frailty, sarcopenia, low muscle strength, stroke, heart disease, and arthritis.

**Results:**

The analysis included 198 patients (mean age 75.9 ± 5.7 years, male 60.1%). After adjusting for potential confounders, depression was associated with TMIG-IC (Prevalence ratio (PR) 2.34, 95% confidence interval (CI) 1.44–3.82), low muscle strength was associated with IADL (PR 2.85, 95% CI 1.30–6.27), and frailty was associated with intellectual activity (PR 1.38, 95% CI 1.10–1.74). In the model with social networks added as a confounder, the relationship between depression or low muscle strength and higher-level functional status was not statistically significant.

**Conclusion:**

Comorbidities of depression and low muscle strength for older patients with type 2 diabetes mellitus increase the risk of malfunctioning of higher-level functional status. Increased interactions with family, friends and neighbours may reduce this event.

**Supplementary Information:**

The online version contains supplementary material available at 10.1007/s41999-024-00937-8.

## Key summary points

**Aim:**

To investigate the association between comorbidities associated with diabetes and higher-level functional status in older patients with type 2 diabetes mellitus.

**Findings:**

Comorbidities of depression, low muscle strength, and physical frailty were associated with the malfunction status of higher-level functional status in older patients with type 2 diabetes mellitus. Expanding social networks reduced the impact of comorbidities on higher-level functional status deterioration.

**Message:**

Care for depression, low muscle strength, and physical frailty, and expanding social networks are important to delay the development of future disabilities for older patients with type 2 diabetes mellitus.

**Supplementary Information:**

The online version contains supplementary material available at 10.1007/s41999-024-00937-8.

## Introduction

Functional status is a key health indicator for older people [1]. In order from simple to complex Lawton's seven-stage hierarchical model, functional status is characterized as the following: life maintenance, functional health, perception–cognition, physical self-maintenance, instrumental self-maintenance, effectance, and social roles [2]. In this hierarchical model, the three complex functions (instrumental self-maintenance, effectance, and social roles) are called higher-level functional status, which are necessary for the capabilities for leading an active and independent life in the community [3]. Previous studies have reported that the malfunction status of higher-level functional status is associated with an increased risk of stroke, death, and cost of medical care or nursing care [4, 5]. Therefore, maintaining higher-level functional status helps prevent adverse health outcome events in the future and prolongs healthy life expectancy.

In Japan, a super-aging society, approximately one in five adults aged 65 and older has diabetes [6]. Elderly patients with diabetes have various comorbidities [7–10], which may affect higher-level functional status. In fact, a systematic review reported that patients with diabetes have an approximately 50–80% increased risk of disability compared to patients without diabetes [11].

Comorbidities associated with the basic activities of daily living (ADLs) and instrumental activities of daily living (IADLs) have been explored in elderly patients with diabetes mellitus; however, few studies have focused on higher-level functional status. Previous studies have reported a longitudinal relationship between the malfunction status of higher-level functional status and cognitive impairment, and physical inactivity, and a cross-sectional association with low muscle strength [12, 13]. A cross-sectional study examined the relationship between comorbidities associated with diabetes and a decline in social roles, suggesting that diabetic nephropathy, depression, and frailty may affect the decline in social roles [14]. These studies suggest that higher-level functional status is based on physical and psychological/cognitive functions.

However, a previous study investigating the relationship between comorbidities associated with diabetes and higher-level functional status reported two common problems. First, confounders that influenced the relationship between comorbidities associated with diabetes and higher-level functional status (e.g., subjective health, educational condition, and socioeconomic factors) were not sufficiently considered. Among them, the social networks (relationships with friends, acquaintances, and family) have been reported to influence physical, mental, psychological, and higher-level functional status [15–17]. Therefore, social networks may influence the association between comorbidities associated with diabetes and higher-level functional status. Second, evidence regarding the relationship between musculoskeletal problems and higher-level functional status in elderly patients with diabetes is lacking. Impairment of basic ADLs and mortality risk are more closely associated with low muscle strength than with sarcopenia [18]. Low muscle strength may have more adverse effects than sarcopenia on higher-level functional status in elderly patients with diabetes mellitus. It is necessary to clarify the relationship between higher-level functional status and comorbidities associated with diabetes so as to identify comorbidities that warrant enhanced care and preventive interventions for the preservation of higher-level functional status.

Therefore, we aimed to investigate the association of comorbidities associated with diabetes including musculoskeletal disorders with higher-level functional status as well as the relationship between comorbidities associated with diabetes and higher-level functional status when the social network in older patients with type 2 diabetes mellitus is in good condition. We set up the following hypotheses: (i) among comorbidities associated with diabetes, low muscle strength, depression, and cognitive decline are associated with the malfunction status of higher-level functional status in older patients with type 2 diabetes mellitus, and (ii) elderly patients with type 2 diabetes mellitus who have better social networks attenuate the effect of comorbidities associated with diabetes on higher-level functional status.

## Methods

### Study design and participant

This cross-sectional study included 198 outpatients with type 2 diabetes aged 65 years or older who attended the Department of Inter Medicine or Diabetes, Endocrinology, and Metabolism, Kohnodai Hospital, National Centre for Global Health and Medicine (Ichikawa City, Chiba Prefecture, Japan) from June 2022 to March 2023. The exclusion criteria were as follows: having severe cardiovascular or respiratory illness, hyperglycaemic crisis, patients with type 1 diabetes, or diabetic foot with ulceration or gangrene. All patients who agreed to participate in the study completed a questionnaire and underwent blood, urine, physical, and cognitive function tests at the next outpatient visit after inclusion. Additionally, information on demographics and treatment status were collected at the same time. This study was approved by the ethics board of the National Centre for Global Health and Medicine (approval number: NCGM-S-004396-00) and was conducted following the principles of the Declaration of Helsinki. Written informed consent was obtained from all the patients before participating in the study.

### Assessment of higher-level functional status

The self-administered questionnaire of the Tokyo Metropolitan Institute of Gerontology Index of Competence (TMIG-IC) was used to assess higher-level functional status of patients [19, 20]. The TMIG-IC is an index of higher-level functional status commonly used in Japan, which corresponds to Lawton’s hierarchical model of instrumental self-maintenance, effectance, and social roles. TMIG-IC composed of 13 questions. The response to each question was rated as “0” for no and “1” for yes. The total score for the TMIG-IC and each subscale was obtained by adding the scores of all questions (maximum score: IADL = 5; intellectual activity = 4; social role = 4; and TMIG-IC score = 13). A higher score represents good condition of higher-level functional status. Previous studies have reported that TMIG-IC has high reliability and validity [19]. In this study, patients whose total score of TMIG-IC was ≤ 9 were defined as having malfunction status of higher-level functional status [4, 21, 22]. Regarding the subscales, a patient was defined as having malfunction status of each subscale, if they reported not full score (score ≤ 4 out of 5 for IADL; ≤ 3 out of 4 for intellectual activity or social role) [4].

### Assessing of comorbidities associated with diabetes

The following comorbidities associated with diabetes were investigated based on medical records, blood tests, urinalysis, questionnaires, and physical and cognitive functioning tests: diabetic peripheral neuropathy, diabetic retinopathy, diabetic nephropathy, cognitive impairment, depression, physical frailty, sarcopenia, low muscle strength, stroke, heart disease, and arthritis.

Diabetic peripheral neuropathy was defined as meeting at least two of the following three criteria: the presence of subjective symptoms (numbness, pain, and paraesthesia in both soles or toes), reduced Achilles tendon reflex, and decreased vibration sensation in the medial malleolus [23].

Diabetic retinopathy was investigated using ophthalmologists’ medical records within six months. It was classified as no diabetic retinopathy (NDR), simple retinopathy (SDR), pre-proliferative retinopathy (pre-PDR), or proliferative retinopathy (PR) according to Davis’ classification [24].

Diabetic nephropathy was classified into five stages according to the classification of Diabetic Nephropathy 2014, based on the results of urinary albumin/Cr ratio (Albumin Creatinine Ratio: ACR) and estimated glomerular filtration rate (eGFR) (Stage 1: ACR < 30 mg/gCre, Stage 2: ACR is 30–299 mg/gCre, Stage 3: ACR is ≥ 300 mg/gCre, Stage 4: eGFR is < 30 mL/min/1.73 m^2^ regardless of ACR value, and Stage 5: maintenance dialysis) [25]. In this study, the presence of nephropathy was defined as patients with Stage 2 or higher [14].

Cognitive function was evaluated using the Mini-Mental State Examination (MMSE) [26]. The total possible score was 30, with higher scores indicating better cognitive function. Cognitive impairment was defined as an MMSE score ≤ 23 points [27].

Depression was assessed by GDS-15 (Geriatric Depression Scale-15), with higher scores indicating a higher degree of depression [28]. In this study, a GDS score of ≥ 10 was defined as depression referring to the previous study [29].

Physical frailty was defined according to the Japanese version of the Cardiovascular Health Study (J-CHS) criteria [30]. Patients underwent grip strength measurements using a grip strength meter (GRIP-D5401; Takei Scientific Instruments Co., Ltd., Niigata, Japan) [31], a 5-m usual walking test [32], and were asked about weight loss, fatigue, and physical activity. The presence of physical frailty was defined as three or more of the following items: muscle weakness (grip strength: < 28 kg for men, < 18 kg for women), decrease in walking speed (usual walking speed < 1.0 m/s), weight loss (weight loss of ≥ 2 kg in the last 6 months), fatigue (feeling unexplainedly tired for the last 2 weeks), and inactivity (Do you do light exercise at least once a week? Do you participate in regular sports at least once a week? If they answered “no” to both questions).

Sarcopenia and low muscle strength were classified based on the AWGS2019 [33]. Sarcopenia was defined as decreased muscle strength (grip strength: < 28 kg for men, < 18 kg for women) and decreased skeletal muscle mass index (SMI) according to the Bioelectrical Impedance Analysis (BIA) method (SMI: < 7.0 kg/m^2^ for men, < 5.7 kg/m^2^ for women). Low muscle strength was defined as decreased muscle strength (grip strength: < 28 kg for men, < 18 kg for women), but normal SMI (SMI: ≥ 7.0 kg/m^2^ for men, ≥ 5.7 kg/m^2^ for women).

To investigate the presence of stroke (cerebral infarction or hemorrhage), cardiovascular disease (myocardial infarction, angina pectoris, and heart failure), and arthritis, the patients were asked, “have you ever been diagnosed or treated before?” for each disease. If they answered “yes,” they were defined as having comorbidities from each disease.

### Other variables

We also collected the following information for using as covariates referencing previous studies [12, 13, 34]: age, sex, body mass index (BMI), insulin use status (using insulin or not), HbA1c level, smoking status (current smoker or not), educational status (less than high school education, high school education, or more than high school education), subjective economic conditions (poor/normal or good), subjective health (healthy or not), living status (living alone or not), physical activity, and social networks. The body mass index (BMI) was calculated by dividing the patient’s weight in kilograms by the square of their height in meters (kg/m^2^) and classified as underweight (BMI < 18.5), normal weight (18.5 ≤ BMI < 25.0), and obese (BMI ≥ 25.0). Physical activity was assessed by the Physical Activity Scale for the Elderly (PASE) [35]. PASE assessed the physical activities of three domains (leisure, household, and work-related activity) for the past week. Higher scores indicate better physical activity. To assess social networks, we used the Lubben Social Network Scale-short version (LSNS-6) [36]. LSNS-6 consists of six questions on interactions with family and friends. The scores range from 0 to 30, with higher scores indicating larger social networks.

### Statistical analysis

Continuous variables were expressed as the mean ± standard deviations (SD), and categorical variables as ratios. To investigate the relationship between the malfunction status of higher-level functional status and comorbidities associated with diabetes, Poisson regression analysis was conducted, and the prevalence ratio (PR) and 95% confidence interval (CI) were calculated for each related factor. The malfunction status of the TMIG-IC or each subscale was used as an objective variable. Comorbidities associated with diabetes were used as explanatory variables, which were individually put into Poisson regression analysis. The use of logistic regression analysis for data with a high frequency of outcomes results in the gap between the calculated odds ratio and the true relative risk value widening. Therefore, if the frequency of the outcome (patients who had malfunction status of higher-level functional status) is > 10%, as in this study, calculation of prevalence ratios using Poisson regression analysis is recommended [37, 38]. Therefore, we conducted Poisson regression analysis. Cognitive impairment, diabetic nephropathy, depression, physical frailty, and low muscle strength were considered as comorbidities associated with diabetes. These comorbidities associated with diabetes have been reported to be associated with higher-level functional status [12, 13, 39]. A supplemental analysis was conducted to investigate the relationship between other comorbidities associated with diabetes (diabetic peripheral neuropathy, sarcopenia, stroke, heart disease, and arthritis) and higher-level functional status. Additionally, we adjusted for confounders using propensity scores, which allowed multiple confounders to be aggregated into one variable. The effectiveness of controlling for confounders using propensity scores has already been reported in several observational studies [40, 41]. The propensity scores used for the analysis in this study included the following variables: age, sex, BMI, insulin use status, HbA1c, smoking status, educational status, subjective economic condition, subjective health, living status, physical activity, and other comorbidities associated with diabetes, which were not included as explanatory variables. Thus, we calculated the propensity scores for each comorbidity associated with diabetes in this study separately. Model 1 included the abovementioned propensity scores as moderating variables. Furthermore, to investigate the impact of social networks on the association between comorbidities associated with diabetes and higher-level functional status, Model 2 included LSNS-6 scores as moderator variables in addition to Model 1.

All statistical analyses were conducted using SPSS IBM SPSS (Version 28.0; IBM Corp., Armonk, NY, USA). In this study, we investigated the association between higher-level functional status individually for each comorbidity associated with diabetes (five items); therefore, it was necessary to consider the multiplicity of the test. Thus, Bonferroni correction (0.05/5 = 0.01) was conducted, and the significance level was set at *P* < 0.01.

## Results

The descriptive characteristics of the 198 patients who participated in this study are shown in Table 1. The mean ± SD age of the patients was 75.9 ± 5.7 years, and 119 (60.1%) were male. The mean ± SD of the TMIG-IC score was 10.7 ± 2.2 points, and 49 patients (24.9%) had TMIG-IC malfunction status. The mean ± SD of scores for IADL, intellectual activity, and social role were 4.7 ± 0.8, 3.2 ± 0.9, and 2.7 ± 1.3 points, respectively. Among the patients, 27 (13.6%), 105 (53.0%), and 126 (64.0%) had malfunctioning status of IADL, intellectual activity, and social roles, respectively.

**Table 1:** Characteristics of the participants (*n* = 198)

Variables	*n*	
Age (years), mean ± SD	198	75.9 ± 5.7
Male sex	198	119 (60.1)
BMI (kg/m^2^)	198	
< 18.5		5 (2.5)
18.5–24.9		113 (57.1)
≥ 25		80 (40.4)
Poor subjective hearing	198	85 (42.9)
Poor subjective visual acuity	198	104 (52.5)
Experience of falling in the past year	198	58 (29.3)
Living alone	198	45 (22.7)
Poor economic condition	198	45 (22.7)
Poor self-rated health	198	32 (16.2)
Latest academic background	198	
Junior high school		23 (11.6)
High school		80 (40.4)
University or vocational college		95 (48.0)
Smoking		23 (11.6)
Physical activity (PASE) (points), mean ± SD	198	103.5 ± 66.6
Social network (LSNS-6) (points), mean ± SD	198	12.8 ± 5.9
Use of medication		
Insulin	198	40 (20.2)
Sulfonylureas	198	19 (9.6)
Biguanides	198	111 (56.1)
Thiazolidines	198	27 (13.6)
α-Glucosidase inhibitors	198	28 (14.1)
Glinides	198	17 (8.6)
DPP4 inhibitors	198	116 (58.6)
SGLT2 inhibitors	198	100 (50.5)
GLP-1 receptor agonists	198	44 (22.2)
HbA1c (%), mean ± SD	197	7.1 ± 0.9
Neuropathy	198	71 (35.9)
Retinopathy	170	45 (22.7)
Nephropathy	197	88 (44.4)
Stroke	198	19 (9.6)
Cardiovascular disease	198	25 (12.6)
Physical frailty	198	49 (24.7)
Depression	195	18 (9.1)
Cognitive decline	198	30 (15.2)
Arthritis	198	10 (5.1)
Sarcopenia	197	18 (9.1)
Low muscle strength	197	55 (27.9)
Higher-level competence		
TMIG-IC score (points), mean ± SD	197	10.7 ± 2.2
IADL score (points), mean ± SD	198	4.7 ± 0.8
Intellectual activity score (points), mean ± SD	198	3.2 ± 0.9
Social role score (points), mean ± SD	197	2.7 ± 1.3
Malfunction status of TMIG-IC	197	49 (24.9)
Malfunction status of IADL	198	27 (13.6)
Malfunction status of intellectual activity	198	105 (53.0)
Malfunction status of social role	197	126 (64.0)

Values are numbers (percentages) unless stated otherwise

*SD* standard deviations, *BMI* body mass index, *PASE* Physical Activity Scale for the Elderly, *LSNS-6* Lubben Social Network Scale-6, *HbA1c* hemoglobin A1c, *TMIG-IC* Tokyo Metropolitan Institute Gerontology Index of Competence, *IADL* Instrumental activities of daily living

The association between the five comorbidities associated with diabetes and the malfunction status of the TMIG-IC are presented in Table 2. In the crude model, the prevalence of TMIG-IC malfunction status was significantly higher in patients with physical frailty, depression, cognitive decline, and low muscle strength (PR 2.46, 5.05, 2.09, and 2.36, respectively). However, these associations were weakened in Model 1, which was adjusted for propensity scores with multiple covariates, and only depression was significantly associated with the malfunctioning status of TMIG-IC (PR2.34). In Model 2, which added LSNS-6 scores to Model 1, there was no significant association between depression and the malfunction status of the TMIG-IC. Incidentally, there was no significant association between other comorbidities associated with diabetes and the malfunction status of the TMIG-IC in any model (Online Resource: Supplementary Table 1).

**Table 2:** Prevalence ratio (95% confidence interval) for malfunction status of TMIG-IC: determined by Poisson regression analysis

Variables	*n*	Prevalence of TMIG-IC disability, *n* (%)	Crude model	Model 1	Model 2
			PR (95% CI)	PR (95% CI)	PR (95% CI)
Diabetic nephropathy	88	27 (30.7)	1.51 (0.93–2.45)	1.28 (0.84–1.94)	1.51 (1.00–2.26)
Physical frailty	49	22 (44.9)	2.46* (1.55–3.90)	1.73 (1.14–2.64)	1.64 (1.10–2.45)
Depression	18	16 (88.9)	5.05* (3.53–7.23)	2.34* (1.44–3.82)	1.59 (1.04–2.44)
Cognitive decline	29	13 (44.8)	2.09* (1.27–3.44)	1.58 (1.02–2.45)	1.78* (1.17–2.71)
Low muscle strength	55	22 (40.0)	2.36* (1.42–3.92)	1.48 (0.94–2.33)	1.40 (0.92–2.15)

Model 1: adjusted for propensity score (including age, gender, use of insulin, HbA1c, BMI, self-rated health, smoking status, physical activity level, academic history, economic condition, living status, other comorbidities). Model 2: Model 1 + score of Lubben Social Network Scale-6

*TMIG-IC* Tokyo Metropolitan Institute Gerontology Index of Competence, *PR* prevalence ratio, *CI* confidence interval

*P < 0.01 (Bonferroni correction was used to adjusted the level of significance from 0.05 to 0.05/5 = 0.01)

The association between comorbidities associated with diabetes and IADL malfunction status are demonstrated in Table 3. In the crude model, the prevalence of malfunction status of IADL was significantly higher in those with depression, cognitive decline, and low muscle strength (PR 3.44; 3.85, and 4.55, respectively). In Model 1, depression and low muscle strength were significantly associated with IADL malfunction status (PR 2.47; 2.85). However, these comorbidities were not significantly associated with the IADL malfunction status in Model 2. There was no significant association between other comorbidities associated with diabetes and IADL malfunction status in any of the models (Online Resource: Supplementary Table 2).

**Table 3:** Prevalence ratio (95% confidence interval) for malfunction status of IADL: determined by Poisson regression analysis

Variables	*n*	Prevalence of IADL disability, *n* (%)	Crude model	Model 1	Model 2
			PR (95% CI)	PR (95% CI)	PR (95% CI)
Diabetic nephropathy	88	15 (17.0)	1.55 (0.77–3.13)	1.77 (0.92–3.42)	1.78 (0.93–3.42)
Physical frailty	49	11 (22.4)	2.09 (1.04–4.20)	0.88 (0.47–1.67)	0.78 (0.42–1.45)
Depression	18	7 (38.9)	3.44* (1.69–7.01)	2.47* (1.30–4.71)	1.90 (0.89–4.05)
Cognitive decline	30	11 (36.7)	3.85* (1.99–7.47)	1.31 (0.60–2.89)	1.41 (0.65–3.05)
Low muscle strength	55	16 (29.1)	4.55* (2.07–9.99)	2.85* (1.30–6.27)	2.56 (1.15–5.68)

Model 1: adjusted for propensity score (including age, gender, use of insulin, HbA1c, BMI, self-rated health, smoking status, physical activity level, academic history, economic condition, living status, other comorbidities). Model 2: Model 1 + score of Lubben Social Network Scale-6

*IADL* instrumental activities of daily living, *PR* prevalence ratio, *CI* confidence interval

*P < 0.01 (Bonferroni correction was used to adjusted the level of significance from 0.05 to 0.05/5 = 0.01)

Table 4 presents the association between comorbidities associated with diabetes and the malfunction status of intellectual activity. In the crude model, patients with depression had a significantly higher prevalence of the malfunction status intellectual disability (PR 1.70), but Models 1 and 2 did not show this association. The crude model showed no significant association between physical frailty and the malfunction status of intellectual activity; however, models 1 and 2 showed a significant association (PR 1.38; 1.35). Incidentally, there was no significant association between other comorbidities associated with diabetes and the malfunction status of intellectual activity in any model (Online Resource: Supplementary Table 3).

**Table 4:** Prevalence ratio (95% confidence interval) for malfunction status of intellectual activity: determined by Poisson regression analysis

Variables	*n*	Prevalence of intellectual activity disability, *n* (%)	Crude model	Model 1	Model 2
			PR (95% CI)	PR (95% CI)	PR (95% CI)
Diabetic nephropathy	88	44 (50.0)	0.91 (0.69–1.19)	0.80 (0.63–1.03)	0.82 (0.64–1.05)
Physical frailty	49	33 (67.3)	1.39 (1.08–1.80)	1.38* (1.10–1.74)	1.35* (1.08–1.69)
Depression	18	15 (83.3)	1.70* (1.31–2.19)	1.11 (0.86–1.43)	1.02 (0.79–1.32)
Cognitive decline	30	20 (66.7)	1.32 (0.98–1.77)	1.31 (1.00–1.72)	1.30 (0.99–1.70)
Low muscle strength	55	35 (63.6)	1.24 (0.96–1.62)	1.01 (0.79–1.30)	1.00 (0.78–1.27)

Model 1: adjusted for propensity score (including age, gender, use of insulin, HbA1c, BMI, self-rated health, smoking status, physical activity level, academic history, economic condition, living status, other comorbidities). Model 2: Model 1 + score of Lubben Social Network Scale-6

*PR* prevalence ratio, *CI* confidence interval

*P < 0.01 (Bonferroni correction was used to adjusted the level of significance from 0.05 to 0.05/5 = 0.01)

The associations between comorbidities associated with diabetes and the malfunction status of social role are shown in Table 5. In the crude model, patients with depression had a significantly higher prevalence of the malfunction status of social role (PR1.57), but Models 1 and 2 did not show this significant association. There was no significant association between other comorbidities associated with diabetes and the malfunction status of social role in any model (Online Resource: Supplementary Table 4).

**Table 5:** Prevalence ratio (95% confidence interval) for malfunction status of social role: determined by Poisson regression analysis

Variables	*n*	Prevalence of social role disability, *n* (%)	Crude model	Model 1	Model 2
			PR (95% CI)	PR (95% CI)	PR (95% CI)
Diabetic nephropathy	88	57 (64.8)	1.03 (0.83–1.27)	0.99 (0.80–1.21)	1.04 (0.86–1.26)
Physical frailty	49	34 (69.4)	1.12 (0.89–1.40)	1.01 (0.80–1.27)	0.97 (0.79–1.18)
Depression	18	17 (94.4)	1.57* (1.33–1.85)	1.32 (1.06–1.65)	1.01 (0.83–1.23)
Cognitive decline	29	23 (79.3)	1.29 (1.04–1.61)	1.20 (0.95–1.52)	1.23 (0.97–1.55)
Low muscle strength	55	39 (70.9)	1.17 (0.94–1.46)	1.05 (0.83–1.32)	0.97 (0.79–1.20)

Model 1: adjusted for propensity score (including age, gender, use of insulin, HbA1c, BMI, self-rated health, smoking status, physical activity level, academic history, economic condition, living status, other comorbidities). Model 2: Model 1 + score of Lubben Social Network Scale-6

*PR* prevalence ratio, *CI* confidence interval

*P < 0.01 (Bonferroni correction was used to adjusted the level of significance from 0.05 to 0.05/5 = 0.01)

## Discussion

We investigated the cross-sectional relationship between comorbidities associated with diabetes and higher-level functional status measured using the TMIG-IC in older patients with type 2 diabetes mellitus who visited a general hospital in Japan. After adjusting for potential confounders (excluding social networks), depression was associated with the malfunction status of the TMIG-IC. The results of the subscale analysis confirmed the association between depression or low muscle strength and the malfunction status of IADL, physical frailty, and the malfunction status of intellectual activity. These results support a part of our hypotheses. Additionally, when the social network (LSNS-6 score) was added as a moderator variable, there was no association between depression or low muscle strength and higher-level functional status. As with our other hypotheses, social networks had a buffering effect on comorbidities associated with diabetes (depression and low muscle strength) and the deterioration of higher-level functional status. These findings suggest that older patients with type 2 diabetes mellitus have an increased risk of deterioration of higher-level functional status due to the comorbidity of depression, low muscle strength, and physical frailty. Furthermore, older patients with diabetes with good social networks (who often interact with others) may attenuate the effect of these comorbidities associated with diabetes on higher-level functional status.

Previous studies investigating the relationship between comorbidities associated with diabetes and higher-level functional status had limitations such as insufficient consideration of important confounders (e.g., subjective health, educational conditions, and socioeconomic factors). In this study, the propensity score was used to account for the potential confounders, which may indicate a result with higher internal validity. Additionally, we investigated the relationship between comorbidities associated with diabetes, including musculoskeletal problems, and the hierarchy of higher-level functional status in older patients with type 2 diabetes mellitus. In terms of clinical implications, these findings suggest the comorbidity for which enhanced care and preventive interventions should be recommended to maintain higher-level functional status. In other words, good control of comorbidities such as low muscle strength, frailty, and depression may contribute to preventing a decline in higher-level functional status that leads to future disability.

We have shown that comorbid depression increases the risk of the malfunction status of higher-level functional status in older patients with type 2 diabetes mellitus. Social role as a factor in higher-level functional status has been reported to be associated with depression [14]. Besides, it has been suggested that patients with diabetes with elevated depressive symptoms after the onset of diabetes may develop disability earlier [42]. Therefore, the results of this study are considered valid and support the importance of care and support for depression to prevent future disability at an early stage.

In the subscale analysis, low muscle strength was associated with a significantly higher prevalence of IADL malfunction status. Although there are differences in the definitions of low muscle strength, a previous study of community-based elderly also found an association between low muscle strength and difficulty with IADL [39]. Other previous studies have suggested that muscle strength may predict the deterioration of IADL more accurately than muscle mass [43], and that maintaining higher muscle strength contributes to the prevention of IADL disorders [44]. Therefore, measures to prevent low muscle strength (e.g. exercise and nutritional management) may be more important in older patients with type 2 diabetes mellitus. In addition, older patients with type 2 diabetes mellitus and physical frailty had a significantly higher risk of intellectual activity malfunction. A systematic review by Kojima et al. reported that physical frailty increases the risk of cerebrovascular dementia and Alzheimer's disease [45]. Although the theoretical pathway is unclear, physical frailty (easily fatigable, decreased activity) interferes with engaging in intellectual activities, which may be one of the causes of cognitive decline. In contrast, Abe et al. reported reverse causality, in which older people who engage in intellectual activities are more likely to improve from physical frailty to non-physical frailty [46]. Considering the bidirectional causal relationship between physical frailty and intellectual activity as mentioned above, encouraging not only coping with physical frailty but also intellectual activity itself may be useful in prevention of malfunction for higher-level functional status.

As hypothesized, patients with good social networks were unlikely to have depression or low muscle strength and had better higher-level functional status. In other word, these results suggest that the impact of depression or low muscle strength on higher-level functional status is attenuated in the presence of a good social network. Social interaction has been reported to have a buffering effect on the relationship between stress and physical or psychological health [47]. The present study similarly showed that social interaction mitigates the relationship of depression and low muscle strength with higher-level functional status deterioration. Contrary to our hypothesis, cognitive decline significantly increased the prevalence of higher-level functional status malfunction when social networks were included as moderators. Cognitive function greatly contributes to the maintenance of functional status [48]. Thus, patients with cognitive decline are likely to already have impaired higher-level functional status. Consequently, they may already receive support from others, and the amount of interaction may inevitably increase.

A strength of our study is its detailed investigation of the association between comorbidities associated with diabetes and each hierarchy of higher-level functional status, considering important confounders (subjective health, educational status, and social factors). However, this study has several limitations. First, the participants were limited to outpatients attending a general hospital (single centre), which may have been caused sampling bias in this study. Therefore, the results of this study may not apply to both inpatients and nursing home residents. Second, due to cross-sectional study, the causal relationship between comorbidities associated with diabetes and higher-level functional status remains unclear. Therefore, clarifying this causal relationship requires a longitudinal or intervention studies with a large sample of participants from multiple centres. Third, this study did not consider the dietary intake status. Dietary intake affects blood sugar control and various comorbidities associated with diabetes [49], and its relationship with higher-level functional status has also been reported [50]. In the future, it is necessary to consider the dietary intake status.

## Conclusion

In conclusion, comorbidities of depression, low muscle strength, and physical frailty were associated with the malfunction status of higher-level functional status in elderly patients with type 2 diabetes mellitus. Expanding social networks reduced the risk of depression, low muscle strength, and physical frailty were associated with the malfunction status of higher-level functional status in older patients with type 2 diabetes mellitus. Care or prevention of these comorbidities associated with diabetes is critical to delay the development of future disabilities. Additionally, expanding social networks may reduce the impact of comorbidities such as depression and low muscle strength on higher-level functional status.

## Supplementary Information

Below is the link to the electronic supplementary material.

Supplementary file1 (PDF 180 KB)

## Data Availability

The data that support the findings of this study are available from the corresponding author on reasonable request.
