# Supplementary material for: Association between comorbidities associated with diabetes and higher-level functional status in older patients with type 2 diabetes mellitus: a cross sectional study
Source: Eur Geriatr Med. 2024 Feb 10;15(4):1101–10. doi: 10.1007/s41999-024-00937-8 (PMC11377677; doi:10.1007/s41999-024-00937-8)
Supplement: Supplementary file 1 — Supplementary file1 (PDF 180 KB) [file 41999_2024_937_MOESM1_ESM.pdf]

Supplementary Information

The relationship between other comorbidities associated with diabetes (diabetic peripheral neuropathy, sarcopenia, stroke, heart disease, and arthritis) and higher-level functional status.

Article title

Association between comorbidities associated with diabetes and higher-level functional status in older patients with type 2 diabetes mellitus – A cross sectional study

Journal name

European Geriatric Medicine

Author names and affiliations

Takuro Shoji, PT, MS [1, 2], Kenta Kogure, PT [1], Nagisa Toda, PT [1], Mariko Hakoshima, MD [3], Hisayuki Katsuyama, MD, PhD [3], Hidekatsu Yanai, MD, PhD [3], Satoshi Tokunaga, MS [2], Korin Tateoka, MS [4], Taishi Tsuji, PhD [5], Tomohiro Okura, PhD [5]

[1] Department of Rehabilitation Medicine, National Center for Global Health and Medicine Kohnodai Hospital, Ichikawa, Chiba, Japan

[2] Graduate School of Comprehensive Human Sciences, Doctoral Program in Public Health, University of Tsukuba, Tuskuba, Ibaraki, Japan

[3] Department of Diabetes, Endocrinology, and Metabolism, National Center for Global Health and Medicine, Kohnodai Hospital, Ichikawa, Japan

[4] Graduate School of Comprehensive Human Sciences, Doctoral Program in Physical Education, Health and Sport Sciences, University of Tsukuba, Tuskuba, Ibaraki, Japan

[5] Institute of Health and Sport Sciences, University of Tsukuba, Tuskuba, Ibaraki, Japan

Corresponding author

Takuro Shoji, PT, MS, Department of Rehabilitation Medicine, National Center for Global Health and Medicine Kohnodai Hospital, Kohnodai 1-7-1, Ichikawa, Chiba 272-8516, Japan.

Phone number: +81 47-372-3501

Fax number: +81 47-375-4746

Email address: pt.shoji@hospk.ncgm.go.jp

Supplementary Table 1. Prevalence ratio (95% confidence interval) of other comorbidities for malfunction status of TMIG-IC: determined by Poisson regression analysis

**Variables**	***n***	**Prevalence of TMIG-IC disability, *n* (%)**	**Crude model**	**Model 1**	**Model 2**
			**PR (95% CI)**	**PR (95% CI)**	**PR (95% CI)**
Arthritis	10	3 (30.0)	1.22 (0.46－3.25)	1.27 (0.54－2.96)	0.90 (0.35－2.36)
Diabetic neuropathy	71	15 (21.1)	0.78 (0.46－1.34)	0.79 (0.51－1.24)	0.90 (0.59－1.39)
Stroke	19	5 (26.3)	1.07 (0.48－2.36)	1.10 (0.60－2.04)	0.78 (0.41－1.49)
Cardiovascular disease	25	9 (36.0)	1.55 (0.86－2.79)	0.86 (0.50－1.49)	0.84 (0.50－1.41)
Sarcopenia	18	6 (33.3)	1.38 (0.68－2.79)	1.28 (0.70－2.35)	1.00 (0.47－2.13)

TMIG-IC, Tokyo Metropolitan Institute Gerontology Index of Competence; PR, prevalence ratio; CI, confidence interval.

Model 1: adjusted for propensity score (including age, gender, use of insulin, HbA1c, BMI, self-rated health, smoking status, physical activity level, academic history, economic condition, living status, other comorbidities). Model 2: Model 1 + score of Lubben Social Network Scale-6

Supplementary Table 2. Prevalence ratio (95% confidence interval) of other comorbidities for malfunction status of IADL: determined by Poisson regression analysis

**Variables**	***n***	**Prevalence of IADL disability, *n* (%)**	**Crude model**	**Model 1**	**Model 2**
			**PR (95% CI)**	**PR (95% CI)**	**PR (95% CI)**
Arthritis	10	2 (20.0)	1.50 (0.41－5.48)	1.65 (0.71－3.85)	1.47 (0.63－3.47)
Diabetic neuropathy	71	8 (11.3)	0.75 (0.35－1.63)	0.77 (0.39－1.51)	0.74 (0.37－1.48)
Stroke	19	4 (21.1)	1.64 (0.63－4.24)	1.18 (0.43－3.25)	1.06 (0.39－2.91)
Cardiovascular disease	25	4 (16.0)	1.20 (0.45－3.19)	1.13 (0.51－2.50)	1.08 (0.51－2.27)
Sarcopenia	18	3 (16.7)	1.24 (0.42－3.73)	1.38 (0.61－3.15)	1.44 (0.58－3.55)

IADL, Instrumental activities of daily living; PR, prevalence ratio; CI, confidence interval.

Model 1: adjusted for propensity score (including age, gender, use of insulin, HbA1c, BMI, self-rated health, smoking status, physical activity level, academic history, economic condition, living status, other comorbidities). Model 2: Model 1 + score of Lubben Social Network Scale-6

Supplementary Table 3. Prevalence ratio (95% confidence interval) of other comorbidities for malfunction status of intellectual activity: determined by Poisson regression analysis

Variables	*n*	Prevalence of intellectual activity disability, *n* (%)	Crude model	Model 1	Model 2
			PR (95% CI)	PR (95% CI)	PR (95% CI)
Arthritis	10	3 (30.0)	0.55 (0.21－1.44)	0.52 (0.25－1.09)	0.50 (0.24－1.02)
Diabetic neuropathy	71	39 (54.9)	1.06 (0.81－1.38)	1.03 (0.82－1.30)	1.03 (0.82－1.30)
Stroke	19	12 (63.2)	1.22 (0.84－1.76)	1.12 (0.78－1.61)	1.08 (0.75－1.54)
Cardiovascular disease	25	15 (60.0)	1.15 (0.81－1.64)	0.92 (0.62－1.36)	0.91 (0.63－1.33)
Sarcopenia	18	6 (33.3)	0.60 (0.31－1.17)	0.69 (0.39－1.21)	0.67 (0.38－1.17)

PR, prevalence ratio; CI, confidence interval.

Model 1: adjusted for propensity score (including age, gender, use of insulin, HbA1c, BMI, self-rated health, smoking status, physical activity level, academic history, economic condition, living status, other comorbidities). Model 2: Model 1 + score of Lubben Social Network Scale-6

Supplementary Table 4. Prevalence ratio (95% confidence interval) of other comorbidities for malfunction status of social role: determined by Poisson regression analysis

Variables	*n*	Prevalence of social role disability, *n* (%)	Crude model	Model 1	Model 2
			PR (95% CI)	PR (95% CI)	PR (95% CI)
Arthritis	10	5 (50.0)	0.77 (0.41－1.45)	0.77 (0.42－1.39)	0.69 (0.38－1.23)
Diabetic neuropathy	71	46 (64.8)	1.02 (0.82－1.27)	1.01 (0.81－1.25)	1.02 (0.84－1.24)
Stroke	19	12 (63.2)	0.99 (0.69－1.41)	0.97 (0.67－1.40)	0.80 (0.57－1.12)
Cardiovascular disease	25	15 (60.0)	0.93 (0.66－1.31)	0.78 (0.54－1.13)	0.78 (0.58－1.05)
Sarcopenia	18	12 (66.7)	1.04 (0.74－1.47)	1.05 (0.74－1.47)	1.03 (0.72－1.49)

PR, prevalence ratio; CI, confidence interval.

Model 1: adjusted for propensity score (including age, gender, use of insulin, HbA1c, BMI, self-rated health, smoking status, physical activity level, academic history, economic condition, living status, other comorbidities). Model 2: Model 1 + score of Lubben Social Network Scale-6
